# Transdifferentiation of neutrophils facilitates the establishment of infection by *Leishmania donovani* parasites

**DOI:** 10.1128/iai.00409-24

**Published:** 2025-05-09

**Authors:** Madhurima Roy, Aniruddha Bagchi, Chaitali Karmakar, Mitali Chatterjee

**Affiliations:** 1 Department of Pharmacology, Institute of Postgraduate Medical Education and Research (IPGME&R) 30164 , Kolkata, India; University of California Davis, Davis, California, USA

**Keywords:** apoptosis, neutrophil-dendritic cell (N-DC) hybrids, phagocytosis, Post Kala-azar Dermal Leishmaniasis (PKDL), reactive oxygen species, transdifferentiation

## Abstract

Neutrophil transdifferentiation involves the acquisition of dendritic cell-like properties, challenging the traditional view of neutrophils being solely phagocytes. The presence of transdifferentiated neutrophils is established in Visceral Leishmaniasis, but not in its dermal sequel, Post Kala-azar Dermal Leishmaniasis. Accordingly, this study investigated the altered functionalities of neutrophils focusing on the acquisition of dendritic cell-like properties and its impact on infection establishment. In PKDL cases, immunophenotyping of neutrophil-dendritic cells (N-DC hybrids) was performed using flow cytometry, along with studying the status of N-DC hybrid inducing cytokines (TNF-α, IFN-γ) and growth factor (GM-CSF). *Ex vivo* infection of neutrophils with *L. donovani* was monitored by droplet digital PCR, employing *A2*; additionally, their frequency of transdifferentiation, oxidative and phagocytic status, as well as apoptosis potential were quantified by flow cytometry. Compared with healthy controls, neutrophils from PKDL cases demonstrated a significant upregulation of CD83 positivity, but the frequency of co-stimulation (HLA-DR, CD80/86) was unaltered. PKDL cases demonstrated raised levels of TNF-α and IFN-γ, but GM-CSF remained unchanged. Following *ex vivo* infection of neutrophils, infection was evident at 2 h and was accompanied by CD83 positivity. Furthermore, the CD66b^+^/CD83 vis*-à-vis* CD66b^+^/CD83^-^ subset exhibited heightened generation of reactive oxygen species (ROS), enhanced phagocytosis, and increased apoptosis. Taken together, neutrophils from PKDL cases demonstrated transdifferentiation with the absence of antigen-presenting function. Virulent *Leishmania* induced transdifferentiation in neutrophils, altering their functionalities and facilitating parasite uptake, along with heightened generation of intra-neutrophilic ROS and enhanced apoptosis, which possibly facilitated their engulfment by macrophages, thereby bolstering the “Trojan horse” mechanism of parasite transfer.

## INTRODUCTION

*Leishmania* parasites following a sandfly bite are generally harbored within myeloid cells that include neutrophils, macrophages, and dendritic cells (1, 2), with neutrophils being the foremost to arrive at the infection site (3). Neutrophils have been shown to internalize *Leishmania* parasites and, after parasite engulfment, proposed to play a dichotomous host-protective or parasite-permissive role (2, 4), the latter being a pre-requisite for the establishment of infection. This engulfment of parasite-rich apoptotic neutrophils by macrophages facilitates parasite entry as a “Trojan horse” and ensures their vicarious transfer into macrophages (5, 6).

Other strategies of survival adopted by *Leishmania* within human neutrophils include some *Leishmania*-derived components such as lipophosphoglycan (LPG), tartrate-resistant acid phosphatase, and lipid chemotactic factor as they inhibit the leishmanicidal activities of neutrophils, thereby ensuring parasite survival (7). On the other hand, components of the neutrophil leishmanicidal armamentarium include their phagocytic potential, oxidative burst (generation of reactive oxygen species, ROS), and extrusion of neutrophil extracellular traps (NETs) to name a few (8–10). However, some *Leishmania* species have evolved mechanisms to escape NET-mediated killing by expressing 3′-nucleotidase that digests the DNA scaffold (9).

Neutrophils are implicated to have a functional contribution in adaptive immune responses (11, 12), and in this context, transdifferentiation of neutrophils is a non-traditional functionality, wherein classical neutrophils acquire dendritic cell-like properties expressing markers like CD83, class II MHC, co-stimulatory signals (CD80/CD86), etc., and become neutrophil-dendritic cell hybrids (N-DCs) (13, 14). Studies have demonstrated that human neutrophils, when cultured with granulocyte macrophage-colony-stimulating factor (GM-CSF) and interferon-γ (IFN-γ), expressed MHC II (HLA-DR) and also promoted T cell activation along with the expression of co-stimulatory antigens, CD80 and CD86 (15). In Indian visceral leishmaniasis (VL), a subset of low-density circulatory neutrophils was reported that expressed markers of antigen presentation, including major histocompatibility complex Class II and co-stimulatory molecules CD80 and CD86, with augmented expression of arginase 1 and IL-10 (16, 17). Similar findings were observed in cutaneous leishmaniasis (CL) with HLA-DR positivity in circulatory neutrophils (11). This subset of neutrophils compared with conventional neutrophils exhibited increased activation, degranulation, oxidative burst, and phagocytic capacity (11). These emerging concepts of neutrophil heterogeneity and plasticity challenge the classical views of neutrophils being simply innate immune cells with “phagocytic functionality.” In this regard, the presence of N-DC hybrids, if any, in the chronic phase of kala-azar, that is, Post kala-azar dermal leishmaniasis, PKDL is not established; accordingly, this study aimed to delineate the status of circulatory N-DC hybrids in patients with PKDL and the possible role(s) of transdifferentiation in the pathogenesis of *Leishmania* infection.

## RESULTS

### Study population

The study population included PKDL cases (*n* = 16) having features of the polymorphic (*n* = 5) or macular (*n* = 11) form, with the median age being comparable with healthy controls (Table 1). Overall, PKDL cases showed no gender bias, and age- and gender-matched controls were enrolled from non-endemic areas (from different districts of West Bengal), which was in concordance with previous studies [Table 1, (18)]. The median disease duration and lag period (in years) were comparable with previous studies, being 2 and 9 years, respectively [(19), Table 1]. Regarding their treatment for VL, two could not provide this information; among the remaining 14 cases, 9 (65%) received sodium antimony gluconate (SAG), 4 (28%) Liposomal amphotericin B (LAmB), and 1 (7%) an ayurvedic medication. All patients were ITS-1 PCR-positive, and the parasite load was quantified by kDNA qPCR (Table 1).

**TABLE 1:** Study population^*a*^

Characteristics	Patients with PKDL (*n* = 16)	Healthy controls (*n* = 12)
Age (years)^*b*^	26.50 (22.25–39.00)	28.00 (27.00–31.50)
Sex ratio (M:F)	8:8 (1:1)	5:7 (1:1.4)
Lesion type (Polymorphic: Macular)	5:11	NA
Disease duration (years)^*b*^	2.00 (1.00–4.00)	NA
Interval between VL and PKDL (years)^*b*^	9.00 (2.00–23.00)	NA
Parasite load (parasites/µg genomic DNA)^*b*^	Polymorphic PKDL: 10,281 (2,786–1902,39)	NA
	Macular PKDL: 7,126 (2,173–100,727)	

^*a*^ The study population was enrolled following active and passive surveillance.

^*b*^ Values are stated in median (IQR). M: Male, F: Female, VL: Visceral Leishmaniasis, PKDL: Post Kala Azar Dermal Leishmaniasis, NA: Not applicable.

### Presence of neutrophil-dendritic cell hybrids in patients with PKDL

**Fig 1:**
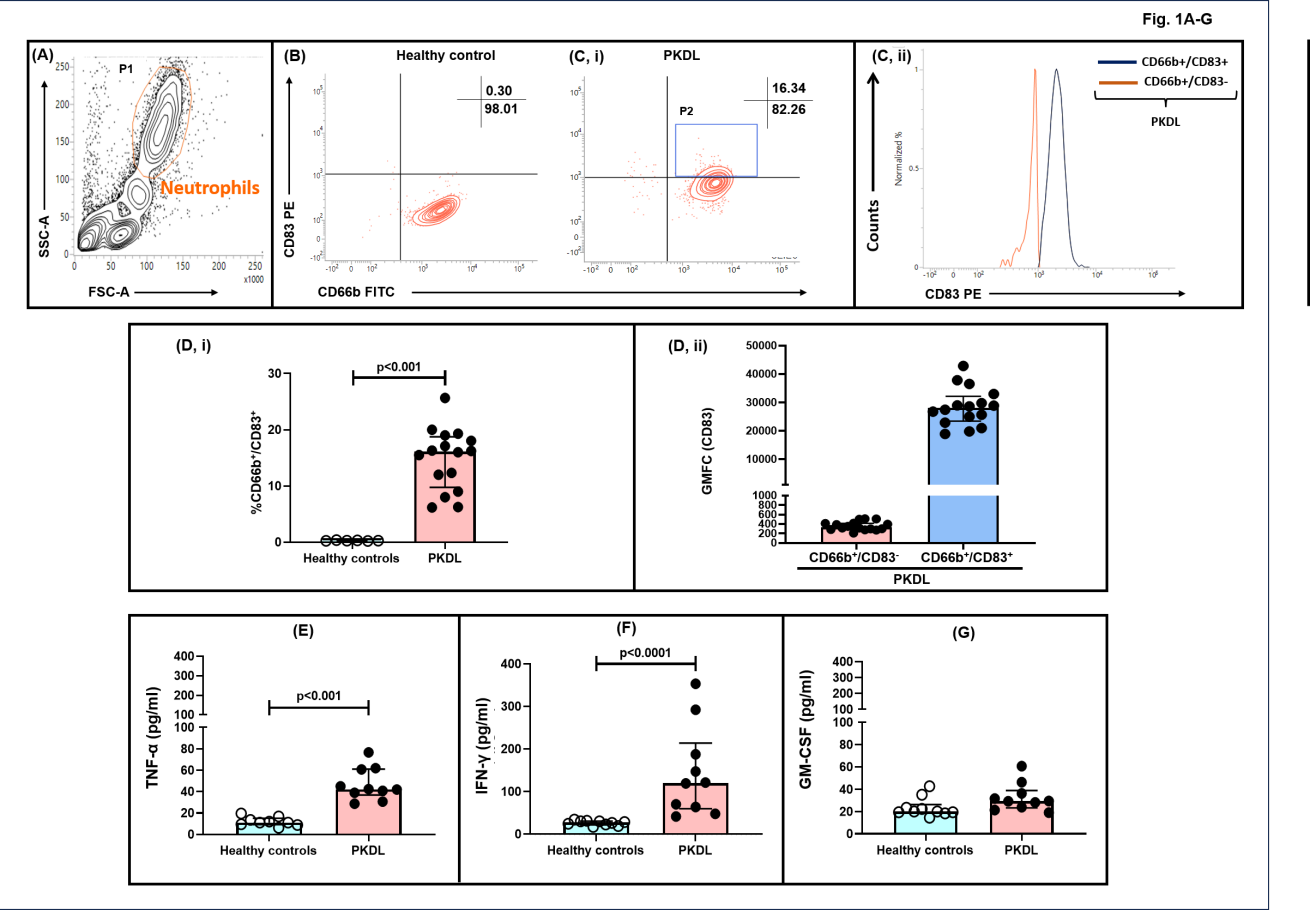
Status of neutrophil-dendritic cell (N-DC) hybrids in PKDL. (**A-C**) Representative profile of neutrophils gated within P1 (**A**) on the basis of forward-scatter (FSC) versus side-scatter (SSC) sourced from peripheral blood of a patient with PKDL. Representative contour plots of the % of CD83 positivity within the CD66b^+^ P1-gated neutrophil population in whole blood sourced from healthy controls (**B**) and a patient with PKDL (**C, I**). Representative histogram plots (**C, II**) and scatter and bar plots (**D, II**) showing expression and median (IQR) GMFC of CD83 within the CD66b^+^/CD83^-^ and CD66b^+^/CD83^+^ neutrophil subpopulations in patients with PKDL. (**D-G)** Scatter and bar plots (**D**) indicating frequency of CD83^+^ within CD66b^+^ cell populations in healthy controls (○) and patients with PKDL (●) along with circulating levels of TNF-α (**E**), IFN-γ (**F**), and GM-CSF (**G**). The horizontal line represents the median; *P* < 0.001, *P* < 0.0001 compared with healthy controls.

In PKDL, neutrophils from whole blood were initially morphologically gated based on FSC-A and SSC-A as “P1” and confirmed with CD66b-fluorescein isothiocyanate (FITC) positivity; within this population, the status of CD83^+^ was examined. The median frequency of the double-positive N-DC hybrids (CD66b^+^/CD83^+^) in PKDL cases compared with healthy controls was substantially enhanced, being 16.27% (10.82%–18.35%) vs. 0.28% (0.24%–0.35%), *P* < 0.001 (Fig. 1A through D). In PKDL cases, with regard to CD83 expression, the CD66b^+^/CD83^+^ (double positive) subset of neutrophils demonstrated an 83.7-fold increase in fluorescence, compared with CD66b^+^/CD83^-^ neutrophils, their median geomean being 28052 (23430–32192) vs. 335 (289–416), respectively (Fig. 1C, ii and D, ii). To understand their co-stimulatory potential, if any, the status of HLA-DR, CD80, and CD86 within the CD66b^+^/CD83^+^ population of neutrophils was measured; with respect to healthy controls, the frequency of all three markers remained unaltered (Table 2). In addition, no significant differences in the expression of CD80/86 were observed within the CD66b^+^/CD83^-^ neutrophil population (Table 2).

**TABLE 2:** Status of co-stimulatory markers within neutrophil dendritic cell hybrids^*a*^

Population(s)	Patients with PKDL [*n* = 16] Frequency (%)	Healthy controls [*n* = 10] Frequency (%)	*P* value
HLA-DR^+^ in CD66b^+^/CD83^+^	0.32 (0.27–0.57)	0.04 (0.00–0.08)	ns
HLA-DR^+^ in CD66b^+^/CD83^-^	0.33 (0.21–0.59)	0.04 (0.00–0.67)	ns
CD80^+^ in CD66b^+^/CD83^+^	0.03 (0.02–0.47)	0.09 (0.04–0.15)	ns
CD80^+^ in CD66b^+^/CD83^-^	0.09 (0.03–0.44)	0.10 (0.09–0.15)	ns
CD86^+^ in CD66b^+^/CD83^+^	0.17 (0.09–0.22)	0.05 (0.01–0.09)	ns
CD86^+^ in CD66b^+^/CD83^-^	0.15 (0.10–0.37)	0.07 (0.04–0.10)	ns

^*a*^ All values in median (IQR). PKDL, Post Kala azar Dermal Leishmaniasis; ns, non-significant.

Before proceeding with whole blood, a sucrose density gradient centrifugation was performed, and the resulting layers included (i) a lower layer consisting of high-density neutrophils (HDNs) positioned above the red blood cells (RBCs) and (ii) an upper lymphocyte layer where low-density neutrophils (LDNs) could potentially reside. However, we did not detect the presence of CD66b^+^ neutrophils in the upper layer, confirming that in PKDL cases, the CD83^+^/CD66b^+^ neutrophils are in the HDN fraction. This suggested that these neutrophils were not classical PMN-DCs, as they lack the distinct expression of MHC-II and co-stimulatory molecules that are typically associated with PMN-DCs.

The cytokine milieu critical for the acquisition of dendritic cell markers and associated co-stimulatory signals in neutrophils includes TNF-α, IFN-γ, and GM-CSF (13, 15, 20). Accordingly, the circulatory levels of TNF-α (Fig. 1E), IFN-γ (Fig. 1F), and GM-CSF (Fig. 1G) were measured. In PKDL cases, compared with healthy controls, the levels of TNF-α were significantly enhanced by 3.80-fold, being (42.34 [30.63–60.76] versus 11.13 [9.15–15.24] pg/ml, *P* < 0.001). Similarly, with IFN-γ, there was a 4.33-fold increase, being (119.00 [47.75–292.30] vs. 27.42 [21.94–30.39] pg/ml, *P* < 0.001), whereas the levels of GM-CSF remained comparable with those of healthy controls (29.37 [23.88–36.26] vs. 20.32 [14.07–26.23] pg/ml) (Fig. 1E–G).

### Establishment of *L. donovani* infection in neutrophils and quantification of parasite load

**Fig 2:**
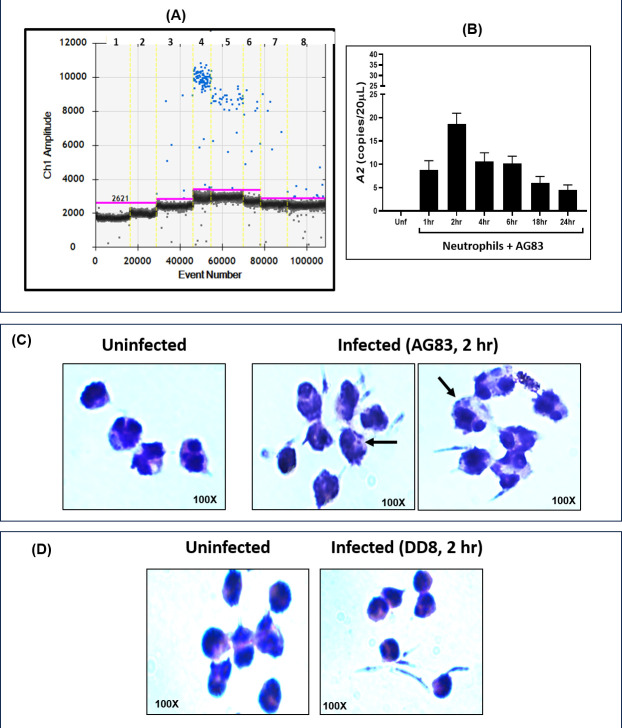
Time kinetics of *Leishmania* infection in neutrophils. (**A-C**) Status of amastigote-specific gene *A2* in *ex vivo L. donovani* (AG83)-infected neutrophils at different time points as measured by ddPCR (**A**) and described in Materials and Methods section. Bar graphs (**B**) indicate the expression of *A2* at different time points and observation of amastigotes (LD bodies) by microscopy following Giemsa staining (**C**). Each horizontal bar represents the median (IQR). Arrows indicate amastigotes within infected neutrophils (**C**). The experiments were repeated thrice in duplicates. (**D**) Giemsa staining of neutrophils following co-incubation with DD8 promastigotes, as observed by microscopy. The absence of LD bodies was confirmed at all time points (1–24 h). The experiments were repeated thrice in duplicates.

In an *ex vivo* model of infection, neutrophils were infected (1–24 h), with virulent, stationary phase *L. donovani* promastigotes (MHOM/IN/83/AG83), and the expression of amastigote-specific gene *A2* was measured by ddPCR (Fig. 2A and B, Table 3). Compared with uninfected neutrophils, *A2* was detected in infected neutrophils, with maximum infection observed at 2 h, the median expression being 19 (13–24) copies/20 µL, following which there was a rapid decline in *A2* (Fig. 2A and B, Table 3); the same was corroborated microscopically (Fig. 2C). Morphologically, the neutrophils harboring the parasites were enlarged, compared with uninfected neutrophils (Fig. 2C).

**TABLE 3:** Time kinetics of infection burden in neutrophils co-incubated with *L. donovani^a^^,^^b^^,c^*

Sets	Time point(s), h	*A2* (copies/20 µL)	*P* value
Neutrophils – *L.d*	Uninfected	0 (0–0)	^*d*^-
Neutrophils + *L.d*	1.0	7 (5–12)	ns
	2.0	19 (13–24)	*P* < 0.01
	4.0	13 (6–15)	ns
	6.0	10 (7–14)	ns
	18.0	7 (2–9)	ns
	24.0	6 (2–7)	ns

^*a*^ Infection of neutrophils by *L. donovani* (AG83) was quantified by measuring *A2* as described in the Materials and Methods.

^*b*^ All values are stated in median (IQR).

^*c*^ ns: non-significant, L.d. *Leishmania donovani*; *P* value, *P* < 0.01 is considered significant compared with infected set at 1 h time point.

^*d*^ "-" defines the IQR range, ie., median value (75 percentile-25 percentile).

Studies suggest that MHOM/IN/80/DD8 (DD8) a *L. donovani* reference strain, usually exhibits lower virulence (21). The co-incubation of neutrophils with avirulent DD8 promastigotes at various time points (1–24 h) failed to demonstrate infection as observed by microscopy, even at the optimal 2 h time point, and their morphology remained comparable with uninfected neutrophils (Fig. 2D).

### Frequency of transdifferentiation in *L. donovani*-infected neutrophils

**Fig 3:**
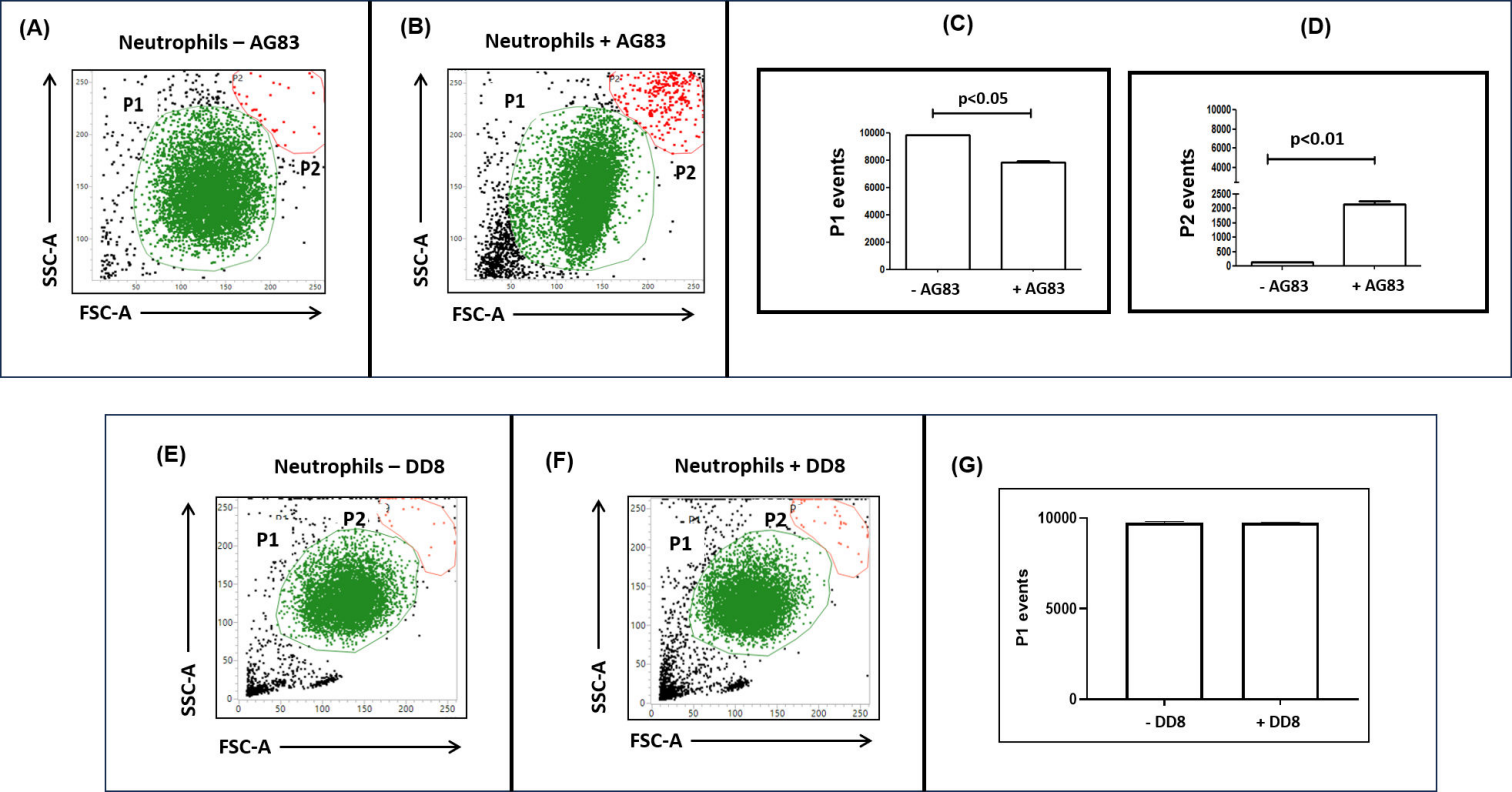
Alterations in morphology of neutrophils following infection with *L. donovani (***A-D**) Representative morphological gating of neutrophils before (**A**) and after infection with *L. donovani* [AG83] (**B**) based on forward (FSC-A) vs. side-scatter (SSC-A) profile as described in the Materials and Methods. Bar graphs indicating the P1 (**C**) and P2 (**D**) events following incubation of neutrophils with *L. donovani* (AG83). Each horizontal bar represents the median (IQR); all experiments were repeated thrice in duplicates; *P* < 0.05 and *P* < 0.01 as compared to uninfected neutrophils. (**E-G**) Representative morphological gating of neutrophils before (**E**) and after incubation with DD8 promastigotes (**F**)**,** based on forward (FSC-A) vs. side-scatter (SSC-A) profiles as described in Materials and Methods. Bar graphs (**G**) indicate the P1 events following the incubation of neutrophils with DD8 promastigotes. Each horizontal bar represents the median (IQR); all experiments were repeated thrice in duplicates.

Following incubation of *Leishmania* parasites (AG83) with neutrophils, infection was maximal at 2 h as quantified by ddPCR (Fig. 2A through C). Morphologically, following infection, the neutrophil population (**P1**) demonstrated an additional population (**P2**), which displayed increased size and granularity (Fig. 3A and B). Irrespective of infection, the median number of events in the P1-gated population remained comparable, being 9,875 (9,828–9,883) and 7,955 (7,531–8,068), respectively (Fig. 3C). However, upon infection, the P2 population demonstrated a substantial increase in the number of events from 95 (99–107) to 2,045 (1,932–2,469), *P* < 0.01 (Fig. 3D). Accordingly, the P2 population was proposed to be the “Trojan horse” subset of neutrophils (6).

The impact of DD8 in modulating the size and granularity following co-incubation with neutrophils was similarly evaluated. In neutrophils co-cultured with DD8, the median P1 events compared with the uninfected scenario were comparable, being 9,743 (9,734–9,768), vs. 9,765 (9,729–9,821), and corroborated with microscopic evaluation (Fig. 2D). Furthermore, unlike the P2 population noted with AG83, there was no distinct P2 population (Fig. 3E through G).

**Fig 4:**
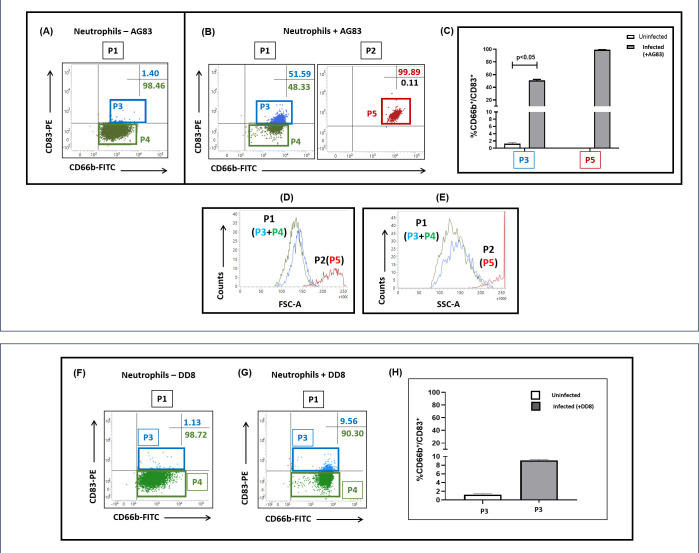
Status of CD83 positivity in neutrophils following exposure to *L. donovani.* (**A, B**) Representative dot plots indicating the frequency [%] of CD66b^+^/CD83^+^ neutrophils ± infection with *L. donovani*. Following infection, the P1 population underwent distribution into P3 and P4 subsets, wherein P3 is CD66b^+^/CD83^+^ and P4 is CD66b^+^/CD83^-^. Upon infection with *L. donovani*, a new P2 subset emerged which, based on CD83 positivity, was gated as P5. (**C**) Bar graphs indicate the % CD83 positivity within the P1/P3 and P2/P5, CD66b^+^ populations in the presence (■, gray box) and absence (□, white box) of *L. donovani* infection. Each horizontal bar represents the median (IQR), *P* < 0.05 is considered significant compared with uninfected neutrophils. (**D, E**) Representative histogram profiles indicating the increased size (**D**) and granularity (**E**) in P2/P5 neutrophils post-infection with *L. donovani* (AG83). All experiments were repeated thrice in duplicates. (**F-H**) Representative dot plots (**F, G**) indicating the frequency [%] of CD66b^+^/CD83^+^ neutrophils in the absence (**F**) and presence (**G**) of DD8 promastigotes. Bar graphs (**H**) indicate the % CD83 positivity within the P1/P3 CD66b^+^ populations in the presence (■, gray box) and absence (□, white box) of DD8 parasites. Each horizontal bar represents the median (IQR).

In AG83-infected neutrophils, the P1 population (Fig. 3A through D) demonstrated two distinct subsets, namely, **P3** (CD66b^+^/CD83^+^ neutrophils) and **P4** (CD66b^+^/CD83^-^ neutrophils) (Fig. 4A and B).

In uninfected neutrophils, the median frequency of CD66b^+^/CD83^+^ transdifferentiated P3 population was 1.36% (1.00%–1.49%) (Fig. 4A), which, following infection, translated into a substantial increase in CD66b^+^/CD83^+^ to 51.05% (49.48%–53.94%)*, P* < 0.05 (Fig. 4B and C). This indicated that > 50% of the P1 neutrophil population, that is, the P1/P3 population acquired CD83 positivity and had become neutrophil-dendritic cells or N-DC hybrids (Fig. 4A through C). This subsequently translated into a decrease in the frequency of the P4, CD66b^+^/CD83^-^ neutrophil subset (Fig. 4A and B). Within the P2 population, which was minimally present in uninfected neutrophils (Fig. 3), infection caused a substantial enhancement of the CD83 positivity in all cells, which were gated as **P5** (CD83^+^/ CD66b^+^) neutrophils (Fig. 4B). The median frequency of this transdifferentiated P5 neutrophil population was 99.00% (99.00%–99.28%) (Fig. 4B and C). Additionally, this P2/P5 subset exhibited the highest expression of CD66b^+^/CD83^+^ with regard to size (FSC-A, Fig. 4D) and granularity (SSC-A, Fig. 4E) *vis-a-vis* P1/P3 and P1/P4, which were comparable (Fig. 4D and E). Taken together, following *L. donovani* infection, the P2/P5 neutrophil subset demonstrated the maximum increase in size, granularity, and expression of CD83.

Infection of neutrophils with DD8 failed to demonstrate any features of transdifferentiation as the baseline transdifferentiation frequency of the P1 neutrophil population was 1.21% (1.08%–1.42%) (Fig. 4F and H) which, post-infection, resulted in a small presence of a CD66b^+^/CD83^+^ (P1/P3) population, majority remaining CD66b^+/^CD83^-^ (P1/P4) population, the median transdifferentiation frequency of the CD66b^+^/CD83^+^ (P3/P1) population being 9.21% (8.15%–9.29%) (Fig. 4G and H). Taken together, compared with AG83, the DD8 promastigotes minimally altered the CD66b^+^/CD83^+^ frequency of the P1/P3 subset (Fig. 4B and C).

### Redox status in infected and transdifferentiated neutrophils

**Fig 5:**
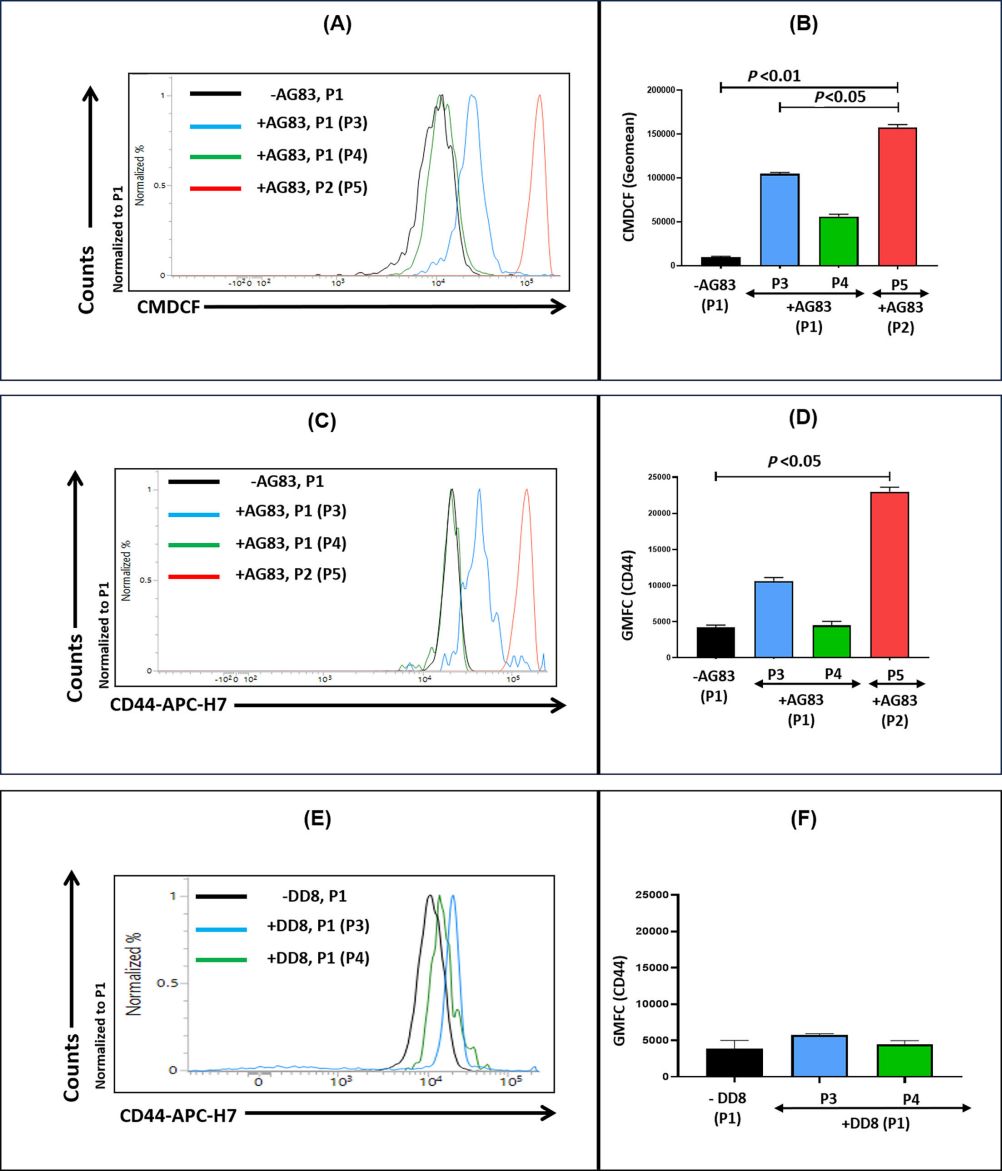
Functional status of neutrophils following infection with *L. donovani.* (**A, B**) Representative histogram profiles (**A**) and bar graphs (**B**) indicating the generation of ROS within neutrophil populations before (P1) and after infection with *L. donovani* (P1/P3, P1/P4) and P2/P5. Each horizontal bar represents the median (IQR), *P* < 0.01 compared with uninfected neutrophils (P1) (**C-F**) Representative histogram profiles (**C, E**) and bar graphs (**D, F**) indicating phagocytic potential within neutrophil populations before (**P1**) and after infection with AG83 [**C, D,** (P1/P3, P1/P4) and P2/P5 neutrophil populations)] or DD8 parasites [**E, F,** (P1/P3 and P1/P4 neutrophil populations)]. Each horizontal bar represents the median (IQR), *P* < 0.05 compared with uninfected neutrophils (P1).

Neutrophils exhibit their defense prowess during infection/inflammation by generating ROS. Accordingly, the impact of virulent *L. donovani* (AG83) infection upon the redox status of neutrophils was evaluated in the P1 and P2 populations. Compared with uninfected neutrophils (P1), whose median geomean fluorescence channel (GMFC) of CM-DCF was 10,224 (7,865–11,483), infection resulted in the generation of two subsets P1/P3 and P1/P4; both showed an enhanced generation of ROS, their median GMFC being 1,04,482 (1,01,033–1,08,023) and 53,688 (50,181–62,512), respectively (Fig. 5A and B). Similarly, the P2/P5 population compared with P1 also exhibited enhanced generation of ROS, being 1,58,165 (1,50,865–1,64,310), *P* < 0.01 (Fig. 5A and B). Taken together, neutrophils that underwent transdifferentiation (CD83^+^) generated a higher proportion of ROS compared with their CD83^-^ subset. However, in neutrophils co-incubated with DD8 promastigotes, the generation of ROS was unaltered compared with the uninfected set (data not shown).

### Status of phagocytic potential in infected and transdifferentiated neutrophil subsets

In view of transdifferentiation being strongly associated with the acquisition of dendritic cell-like properties, its phagocytic potential was assessed. In uninfected neutrophils (P1), the median expression (GMFC) of the phagocytosis marker CD44 was 4,053 (3,616–5,007). Following infection with virulent AG83 parasites, the CD66b^+^/CD83^+^ transdifferentiated neutrophils (P1/P3 and P2/P5) demonstrated an increased phagocytic potential, with the median GMFC being 10,336 (9,772–11,969) and 22,691 (21,883–24,089), *P* < 0.05, respectively (Fig. 5C and D), whereas CD44 expression in the non-transdifferentiated neutrophils (P1/P4) was comparable with uninfected P1 neutrophils, their median expression being 4,763 (3,018–5,786) (Fig. 5C and D). In contrast, the phagocytic potential of neutrophils following incubation with DD8 remained unchanged, as the baseline median GMFC in the P1 subset was 3,912 (3,085–5,007), and post-infection remained comparable in the P3/P1 and P4/P1 populations, being 5,746 (5,184–5,925) and 4,493 (4,088–4,982), respectively (Fig. 5E and F).

### Status of phagocytosis following exposure to CFDA-SE-labeled *Staphylococcus aureus*

**Fig 6:**
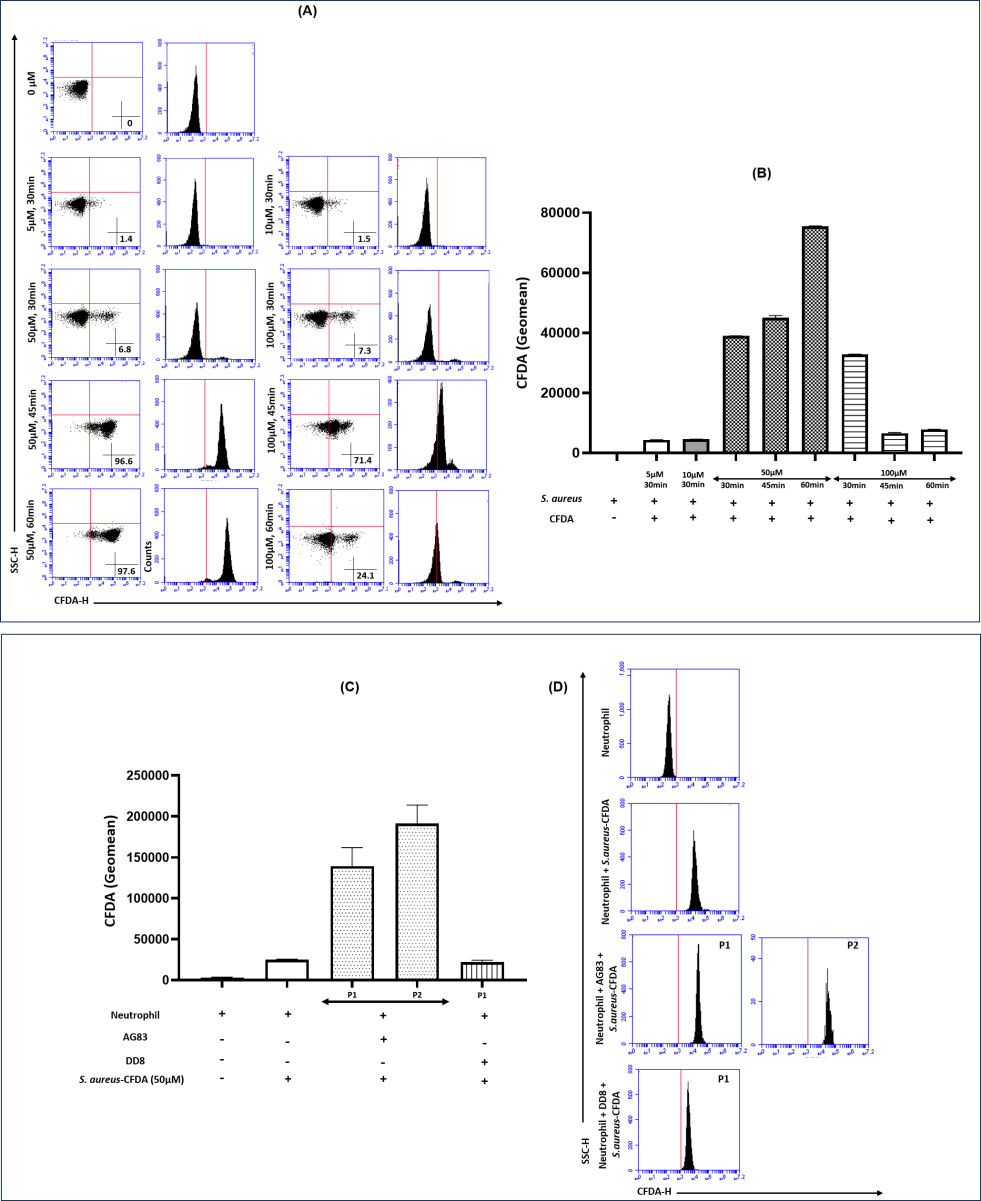
Status of phagocytosis in neutrophils following exposure to CFDA-SE-labeled *S. aureus* (**A, B**) Representative dot and histogram plots (**A**) along with bar graphs (**B**) indicating the optimal CFDA-SE concentration and incubation time for labeling *S. aureus*. Following labeling, purified neutrophils were incubated with either CFDA-SE-labeled *S. aureus* or infected with *L. donovani* promastigotes (virulent AG83 and avirulent DD8) following exposure to *S. aureus*-CFDA to assess phagocytosis. (**C, D**) Bar graphs (**C**) and representative histogram plots (**D**) indicate the CFDA mean fluorescence intensity/geomean as a measure of the relative burden of phagocytosis.

The optimal conditions for labeling *S. aureus* with carboxyfluorescein diacetate succinimidyl ester (CFDA-SE ) were standardized at a concentration of 50 µM for an incubation time of 60 min, resulting in 97.6% of cells exhibiting CFDA uptake (Fig. 6A); their corresponding median GMFC was 75,536 (75,444–75,628, Fig. 6B). The median CFDA GMFC in neutrophils infected with the virulent *L. donovani* AG83, followed by phagocytosis of *S. aureus*-CFDA, was significantly higher, compared with neutrophils exposed only to *S. aureus*-CFDA or those infected with the avirulent strain DD8 before *S. aureus*-CFDA exposure, with the median being 1,25,923 (1,21,521–1,63,703) for P1 population and 1,87,753 (1,71,426–2,12,657) for P2 population vs. 25,164 (24,179–25,389) and 22,444 (19,469–24,245), respectively (Fig. 6C and D). Although the percentage of neutrophils that phagocytosed *S. aureus*-CFDA did not differ significantly across these three groups (data not shown), the CFDA fluorescence intensity/cell (GMFC) was significantly higher in the AG83-infected neutrophils, suggesting that AG83 induced enhanced phagocytosis in infected neutrophils.

### Status of apoptosis in *L. donovani*-infected neutrophils

**Fig 7:**
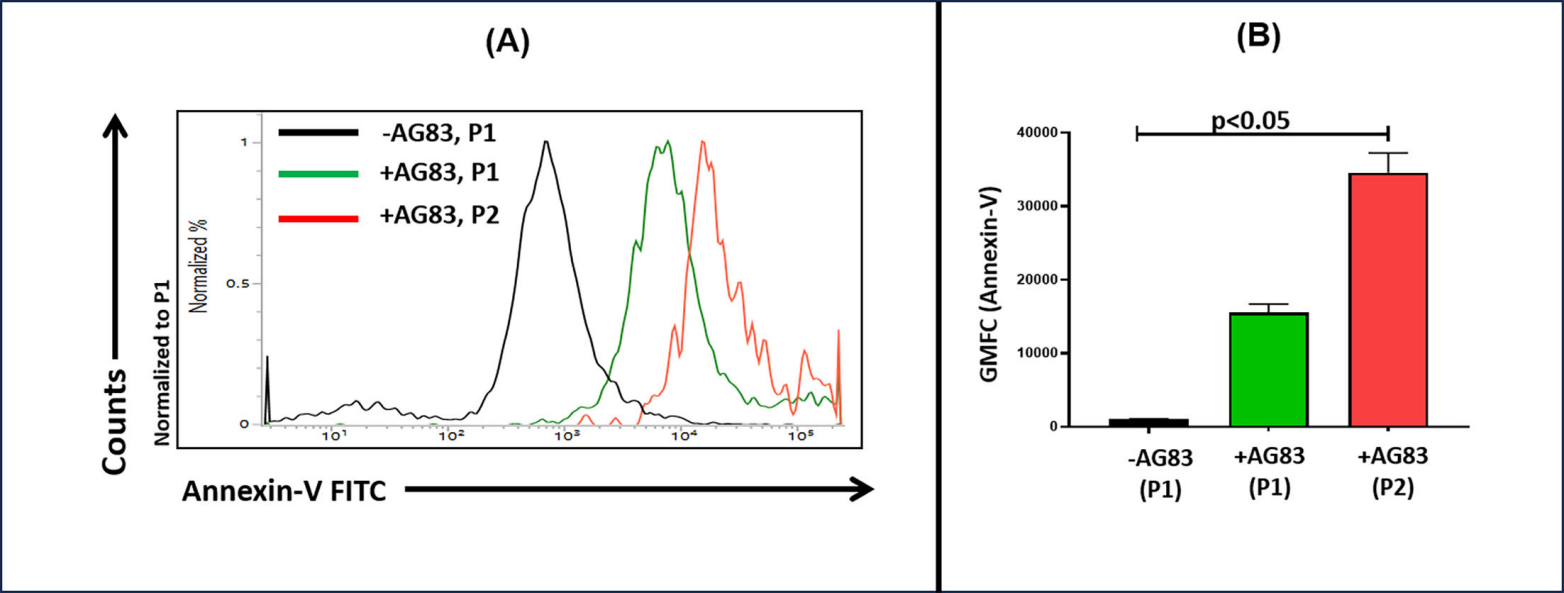
Status of apoptosis in neutrophils following infection with *L. donovani.* (**A, B**) Representative histogram profiles (**A**) and bar graphs (**B**) indicating apoptosis (annexin V^+^) within neutrophil populations before (P1) and after infection with AG83 strain of *L. donovani* (P1/P3, P1/P4) and P2/P5. Each horizontal bar represents the median (IQR), *P* < 0.05 compared with uninfected neutrophils (P1).

*Leishmania* parasites are reported to utilize apoptotic neutrophils as carriers for their covert transmission to host macrophages (6). As infection of neutrophils with virulent promastigotes (Fig. 2A through C) is associated with transdifferentiation (Fig. 4) and increased generation of ROS (Fig. 5A and B), the apoptotic status of these transdifferentiated neutrophil populations was investigated in terms of Annexin V positivity. The populations could not be gated on the basis of CD66b positivity, as Annexin V and CD66b are both FITC-tagged; accordingly, P1 and P2 were gated solely based on their morphological characteristics (FSC-A and SSC-A). Infection with AG83 enhanced apoptosis in P1 and P2 neutrophil populations, the maximal being in the CD66b^+^/CD83^+^ transdifferentiated P2 population (Fig. 7A and B). The median expression of Annexin-V positivity in uninfected neutrophils (P1) was 1,002 (912.5–1,125), which, following infection, increased in the P1 and P2 populations to 15,368 (13,687–17,587) and 32,632 (29,466–40,536), *P* < 0.05, respectively (Fig. 7A and B). On the contrary, DD8 failed to change the apoptotic status of neutrophils (data not shown).

## DISCUSSION

Transdifferentiation of neutrophils results in the generation of N-DC hybrids with dual characteristics, thus combining the immediate microbicidal action of neutrophils with the acquisition of antigen-presenting capabilities of dendritic cells, highlighting the plasticity of immune cells and offering a unique perspective on how diverse immune functions can be integrated to optimize responses to pathogens and overall immune homeostasis (14, 15, 20). This phenomenon was observed in rheumatoid arthritis (RA), wherein the neutrophils in synovial fluid exhibited characteristics akin to dendritic cells, by expressing MHC II and CD83 (13, 14, 22). Similarly, in patients with Wegener’s granulomatosis, neutrophils in peripheral blood demonstrated features of N-DC hybrids, expressing MHC II antigens, indicating a phenotypic shift of classical neutrophils toward antigen-presenting cells (15, 23). Furthermore, in an experimental model of colitis in mice, neutrophils isolated from lesions displayed an upregulation of CD86 alongside MHC II expression, suggesting a potential role in modulating inflammatory responses in the gastrointestinal tract (24). Similarly, in acute bacterial infections, upregulation of CD83 on neutrophils was evidenced (15). In *Leishmania* infections, both cutaneous and visceral, the presence of N-DC hybrids has been reported with the acquisition of HLA-DR and co-stimulatory signals, but their biological relevance remains to be established (11, 17). Taken together, these observations embody the versatile nature of neutrophils in adapting to diverse inflammatory microenvironments, blurring traditional boundaries between innate and adaptive immunity in pathological states and endorsing their putative participation in the pathogenesis of non-pathogen and pathogen-driven diseases.

N-DC hybrids have an enhanced antigen-presenting capability, as they present superantigens to CD4^+^ T cells and activate allogeneic T cells under specific cytokine stimulation [GM-CSF and IFN-γ, (25)]. In an *E. coli*-induced model of infection, N-DC hybrids acquired both MHC class II and co-stimulatory markers, suggesting a role in host defense mechanisms, by efficiently eliminating bacteria via facilitation of enhanced presentation of bacterial antigens to CD4^+^ T cells (26). In mice with chronic colitis, the N-DC hybrids acquired surface markers MHC class II and CD86 that facilitated the induction of OVA-specific CD4^+^ T cell proliferation, suggesting that these hybrid neutrophils potentially perpetuated chronic gut inflammation through T cell activation, proliferation, and increased production of pro-inflammatory cytokines (24). On the contrary, in visceral leishmaniasis, the N-DC hybrids present at disease presentation expressed MHC class II, CD80, and CD86 but were associated with an anergic phenotype (17). Conversely, in CL, the MHC class II/HLA-DR^+^ neutrophils, compared with conventional neutrophils, exhibited increased activation, degranulation, redox status, and phagocytic capability (11). Similarly, in PKDL cases, the maturation marker of classical dendritic cells CD83 was identified within CD66b^+^ neutrophils and was considered as transdifferentiated N-DC hybrids (CD66b^+^/CD83^+^) (Fig. 1). However, these N-DCs showed a conspicuous absence of MHC class II/ HLA-DR and co-stimulatory markers, CD80 and CD86 (Table 2), indicating that these CD83^+^ neutrophils have minimal antigen-presenting function.

PKDL and RA are both chronic inflammatory conditions, with the former being an infectious and the latter being an autoimmune disease; however, both manifest distinct changes in the inflammatory environment at disease presentation (13, 27–29), prompting the investigation into whether the cytokine milieu influenced the differential acquisition of HLA-DR expression in neutrophils. Previous studies have investigated that the interplay of TNF-α, GM-CSF, and IFN-γ plays a pivotal role in the formation of N-DC hybrids (15), wherein the presence of TNF-α, independently or in association with IFN-γ, is responsible for the acquisition of only CD83^+^ in neutrophils. GM-CSF has been reported to be critical for the acquisition of Class II MHC (HLA-DR^+^) and other co-stimulatory signals (CD80^+^ and CD86^+^) in N-DC hybrids (15). As patients with PKDL demonstrated elevated levels of TNF-α and IFN-γ in circulation (Fig. 1), but no changes in the circulating levels of GM-CSF, it possibly accounted for this non-classical N-DC hybrid (Fig. 1, Table 2).

Experimental models of *Leishmania* infection have demonstrated that neutrophils in the initial phase of the disease are the acute responders and play a vital contributory role in the establishment of infection (6). During *L. donovani* infection, neutrophils have been demonstrated to promote parasite survival by hindering host defenses (4) and altering neutrophil functions, such as phagocytosis and apoptosis, to create a favorable niche (30). Furthermore, during the establishment of *L. major* infection, neutrophils have been reported to modulate the dendritic cell functions, wherein the uptake of parasitized neutrophils by dermal DCs diminished the latter’s ability to activate the immune response and delayed host defense mechanisms (7). However, to date, the contribution of neutrophil transdifferentiation to the establishment of infection remains to be investigated. The presence of virulent *L. donovani* within neutrophils confirmed that they can harbor amastigotes (Fig. 2, Table 3) and serve as “non-traditional” host cells. Importantly, this *Leishmania* infection was restricted to virulent promastigotes and led to the generation of an additional neutrophil subset (P2), whose increased size and granularity were attributed to the presence of amastigotes (Fig. 3A through D); the avirulent DD8 strain failed to generate such a P2 population (Fig. 2D; 3E through G). The role of transdifferentiation in facilitating infection was bolstered by the presence of the P2/P5 neutrophil population when infected by virulent AG83, and it showed the highest proportion of transdifferentiation (Fig. 4A through E); these features were notably absent following infection with DD8 (Fig. 4F through H).

The expression of CD83 on neutrophils has been associated with their activation (31). The *L. donovani*-infected CD66b^+^/CD83^+^ neutrophil subsets, that is, P1/P3 and P2/P5, exhibited increased levels of ROS, along with augmented phagocytic function (Fig. 5A through D), in alignment with previous studies that demonstrated transdifferentiation of neutrophils and generation of ROS go hand in hand (13). This would suggest that the N-DC subsets are capable of mounting a potent anti-microbial response against *Leishmania* parasites.

When neutrophils encounter *Leishmania* parasites, there is a production of intracellular ROS secondary to the oxidative burst. Intracellular ROS also serves as a signaling molecule, influencing key processes like inflammation, transdifferentiation, and neutrophil apoptosis; therefore, measuring intracellular ROS provides direct insights into the efficacy of different mechanisms of neutrophil-mediated pathogen elimination, making it an important factor in understanding host-pathogen dynamics. As the extracellular ROS is available once it is released from the cells, it is best suitable to assess the status of ROS that is generated within the cells, that is, intracellular ROS, as this way the measurement additionally identifies the source of ROS.

The heightened production of intracellular ROS, although microbicidal on one hand, renders the neutrophils on the other hand more vulnerable to apoptosis, which facilitates their efficient uptake by host macrophages, serving as efficient conduits for silent transfer of parasites to macrophages (6). In addition, N-DCs retain their phagocytic capabilities and are more efficient than DCs (32, 33). Accordingly, in this study, the transdifferentiated neutrophil subset (P2/P5) infected by AG83 exhibited the highest expression of phagocytic marker CD44, further consolidating the role of CD83 in promoting infection (Fig. 5C and D). The incubation of neutrophils, which were proven to be functionally potent for phagocytosis (Fig. 6A through D), was incubated with DD8 and failed to induce the activation of neutrophils in terms of their redox status, apoptotic potential, and phagocytosis marker expression (Fig. 5E and F; 7A and B). Taken together, the convergence of heightened phagocytosis marker expression and increased apoptosis (Fig. 7A and B) in *L. donovani*-infected neutrophils facilitated the efficient shuttling of parasites to macrophages, reinforcing the “Trojan horse” or “Trojan rabbit” hypothesis. Accordingly, neutrophils can be considered important modulators of Leishmaniasis, and disease intervention strategies should focus on subverting factors that trigger their transdifferentiation.

## MATERIALS AND METHODS

### Reagents

All antibodies were from BD Biosciences (San Jose, CA, USA), and reagents were from Sigma Aldrich (St. Louis, MO, USA), except rK39 immunochromatographic test strips (InBios International, Seattle, WA, USA), QIAmp DNA Mini kit (Qiagen, Hilden, Germany), SYBR Green qPCR Master Mix (Applied Biosystems, Grand Island, NY, USA), ddPCR EvaGreen Supermix (Bio-Rad Laboratories, CA, USA), EasySep Direct Human Neutrophil Isolation Kit (STEMCELL Technologies, VAN, Canada), and Bio-Plex ProTM Human Chemokine Panel 40-plex (Bio-Rad Laboratories, Hercules, CA, USA) for the estimation of IFN-γ, TNF-α and GM-CSF.

### Study population

Patients clinically diagnosed with PKDL (*n* = 16) were recruited following active surveillance-based field surveys (*n* = 13) conducted in Malda and Dinajpur districts of West Bengal, India (18) or by passive surveillance (*n* = 3) from the outpatient department of School of Tropical Medicine (STM), Kolkata. The initial diagnosis was based on clinical features strongly suggestive of PKDL, a prior history of VL, rK-39 positivity, and/or if they resided in an area endemic for VL; subsequently, the diagnosis was confirmed by internal transcribed spacer sequence 1 (ITS-1)-PCR, and parasite load was quantified by qPCR (19). Healthy volunteers (*n* = 15) were recruited from non-endemic and endemic areas and confirmed to be negative for anti-leishmanial antibodies. Heparinized peripheral blood (5 mL) and a dermal biopsy (4 mm) were obtained from a non-cosmetically disfiguring lesional site. From whole blood, immunophenotyping was performed, and plasma was stored for the bioplex assay, whereas dermal biopsies were used for diagnosis and estimation of parasite burden (ITS-1 PCR/qPCR).

### Parasite culture

*L. donovani* (MHOM/IN/83/AG83 and MHOM/IN/80/DD8) promastigotes were maintained at 24°C in M199 medium supplemented with 10% heat-inactivated fetal bovine serum (FBS), penicillin G (50 IU/mL) and streptomycin (50 µg/mL); parasites were sub-cultured every 48–72 h, inoculum being 1 × 10^6^ cells/mL. To obtain virulent promastigotes, BALB/c mice were infected with MHOM/IN/83/AG83; after 45 days, mice were sacrificed, and transformation was achieved in 20–25 days. DD8 promastigotes were rendered avirulent through extensive serial sub-culturing over an extended period of time (>12 weeks).

As parasite virulence is associated with their apoptotic potential (34), the apoptotic status of AG83 and DD8 promastigotes was investigated. In log-phase promastigotes, the baseline apoptosis (early and late apoptosis) of AG83 and DD8 on day 1 was comparable, being 13.70% (10.00%–14.80%) and 7.80% (7.00%–10.00%), respectively (Fig. S2). A repeat evaluation on day 9 of stationary phase promastigotes indicated that in AG83, the proportion of Annexin V positivity was 2.32-fold higher than DD8 promastigotes, being 63.91% (60.30%–65.91%) vs. 27.50% (21.17%–30.09%; Fig. S2).

### Immunophenotyping of neutrophil-dendritic cell (N-DC) hybrids by flow cytometry

Whole blood (100 µL) was stained with anti-human FITC-conjugated CD66b, MHC II cell surface receptor HLA-DR-Peridin-Chlorophyll- protein (PerCP), CD83-Phycoerythrin (PE), co-stimulatory signal CD80-Brilliant Violet (BV510), and CD86-Phycoerythrin (PE)-Cy7. Following a 20-min incubation at RT (20–25°C), erythrocytes were lysed by incubating in BD Fix-lyse RBC lysing buffer for 10 min (2 mL) in dark at RT, washed twice with phosphate-buffered saline (PBS, 0.02M, pH 7.2–7.4), and finally resuspended in PBS (400 µL) for acquisition in a flow cytometer (BD FACSVerse).

### Determination of circulating transdifferentiation-inducing cytokines and chemokines in patients with PKDL

Plasma levels of cytokines responsible for inducing transdifferentiation in neutrophils [TNF-α, IFN-γ, and GM-CSF (15)] were measured (diluted 1:4), using a multiplex detection kit (BioRad, Hercules, CA, USA) as per the manufacturer’s protocol. Data were acquired in a Luminex 200 Labmap system (Luminex, Austin, TX, USA) and analyzed using Bio-Plex Manager software version 6.2.

### Quantification of *Leishmania* infection in neutrophils

Neutrophils were isolated from peripheral blood sourced from healthy donors using the EasySep Direct Human Neutrophil Isolation Kit (STEMCELL Technologies, VAN, Canada) according to the manufacturer’s instructions. Briefly, blood was incubated using RapidSpheres and an isolation cocktail, followed by isolation of neutrophils by negative selection method (to avoid activation) using a magnetic sorter. This step was repeated, followed by two consecutive washes to obtain the enriched cell suspension containing neutrophils. Cell viability (>95%) was confirmed using trypan blue.

For *ex vivo* infection studies, to maintain the purity of neutrophils without prior sorting using the CD66b marker, the EasySep magnetic negative selection method was employed. This approach ensured two key outcomes: (i) the isolation of total and purified neutrophils for infection setup, and (ii) the preservation of their functional inactivity prior to infection (due to negative selection). Consequently, any subsequent changes in the functional parameters post-infection, such as ROS production, phagocytosis, or apoptosis, can be attributed solely to the infection with parasites.

Neutrophils isolated from healthy volunteers were infected with stationary phase *L. donovani* promastigotes, with a parasite:neutrophil ratio of 5:1 at 37°C, 5% CO_2_ for 1–24 h, and the optimal infection time point was monitored. At each time point, the total RNA was isolated by the Trizol method, concentration measured in a NanodropTM One/OneC Microvolume UV-Vis spectrophotometer (Thermo Fischer Scientific, MA, USA), and RNA was converted to cDNA (1 µg per 20 µL reaction) using the high-capacity cDNA reverse transcription kit (Applied Biosystems, MA, USA), according to the manufacturer’s instructions. Detection of amplicons was done using primers (sourced from NCBI Primer-BLAST, https://www.ncbi.nlm.nih.gov/tools/primer-blast) whose specificity was previously confirmed by UCSC *in silico* PCR for amastigote-specific genes *A2* [F, 5′-CTGCAGGCTGTTGACGTTTC-3′; R, 5′-AAGGTTTGCCTCGTCACCAT-3′, (35)].

Briefly, the optimized reaction mixture containing cDNA (50 ng) was added to a ddPCR EvaGreen Supermix (Bio-Rad Laboratories, CA, USA) containing 100 nM/mL of each forward and reverse primer, with the final volume being 20 µL. For negative controls (NC), 10 µL nuclease-free water was added along with 10 µL of ddPCR Eva Green Super mix, and in the case of non-template control (NTC), nuclease-free water was used instead of cDNA. Each reaction was then loaded into a sample well of an 8-well disposable cartridge (DG8), along with 70 µL of droplet generation oil. Droplets were formed using QX200 droplet generators, which were then transferred to a 96-well PCR plate to perform PCR of 40 cycles at 95°C for 5 min, 95°C for 30 s, annealing 58°C for 1 min, with a final extension at 90°C for 5 min. The resultant products were scanned on a QX200 Droplet Reader, and data were analyzed using QuantaSoft software (version 1.7.4, Bio-Rad Laboratories; [35]) and expressed as copies/20 µL with confidence intervals of 95%. The threshold was adjusted based on clear demarcation of positive droplets from the negative cluster with respect to individual wells. The background signals were eliminated using an NC and NTC.

### *Ex vivo* infection of neutrophils with *L. donovani* and status of neutrophil transdifferentiation

Neutrophils were incubated for 2 h with stationary phase *L. donovani* promastigotes (MHOM/IN/83/AG83 and MHOM/IN/80/DD8) in the ratio of 5:1; following centrifugation (8,000 rpm for 3 min) and washing; the cells were surface stained for markers of neutrophil-dendritic (N-DC) cell hybrids (CD66b-FITC and CD83-PE positivity) and acquired in a flow cytometer.

### Measurement of ROS in infected neutrophils

*Leishmania*-infected/uninfected neutrophils (5 × 10^5^ /mL) were incubated with CM-H_2_DCFDA (2 µM) for 30 min at 37°C in dark; after two washes with PBS, the cells were resuspended in 400 µL of PBS and acquired in a flow cytometer. CM-H_2_DCFDA is a non-fluorescent probe, which, upon enzymatic hydrolysis by cellular esterase and oxidation by intracellular ROS, produces a fluorescent compound chloromethyl dichloro-fluorescein (CM-DCF). This CM-DCF-based fluorescence is directly proportional to the amount of intracellular ROS and was measured in a flow cytometer.

### Measurement of phagocytic potential (CD44) in infected neutrophils

Infected/uninfected neutrophils with either AG83 or DD8 were surface-stained with a marker for phagocytosis (CD44-APC-H7) for 30 min at 37°C in dark (as per manufacturer’s instructions); after two washes with PBS, the cells were resuspended in 400 µL of PBS and acquired in a flow cytometer. The frequency of CD44 on neutrophils in both uninfected and infected groups was 100%, that is, all neutrophils had CD44 as their surface marker. Therefore, the expression per cell (neutrophil) was measured instead of the frequency and expressed in terms of GMFC (geomean of fluorescence intensity).

### Annexin-V binding assay

Apoptosis in neutrophils was determined by Annexin V- FITC binding according to the manufacturer’s recommendations. Briefly, neutrophils (5 × 10^5^ /mL) after incubation with *L. donovani* for 2 h were subsequently washed two times in PBS and resuspended in Annexin V binding buffer (100 µL, 10 mM HEPES, pH 7.4, 140 mM NaCl, 5 mM CaCl_2_) for 20 min in the dark at 37°C. The cells were then stained with FITC-labeled Annexin-V for 20 min in the dark at RT, washed, resuspended in binding buffer, and acquired in a flow cytometer. Data were expressed as geomean (expression) in P1 and P2 neutrophil subsets.

To evaluate the degree of apoptosis in *Leishmania* promastigotes (MHOM/IN/83/AG83 and MHOM/IN/80/DD8), dual staining for Annexin V-FITC and Propidium iodide (PI) was performed as previously described (36). Briefly, according to the manufacturer’s instructions, promastigotes (1 × 10^6^ /mL) at day 1 and day 9 (stationary phase) were incubated in Annexin V binding buffer for 10 min, followed by incubation with Annexin V-FITC and PI (0.05 µg/mL) for 30 min in the dark at 24°C. Hydrogen peroxide (H_2_O_2_, 20 mM, 2 h, 37°C) served as a positive control for Annexin V, and heat-killed cells as a positive control for PI. Data acquisition was done on a flow cytometer. The % Annexin V positivity included the % gated cells in the right lower and upper quadrant to represent early and late apoptosis, respectively.

### Labeling of *S. aureus* and phagocytosis assay

The ability of neutrophils to phagocytose fluorescent particles was quantified by flow cytometry. A stock solution of 2 mM CFDA-SE (carboxyfluorescein diacetate succinimidyl ester) was prepared by dissolving 0.92 mg of CFDA in 1 mL of anhydrous DMSO, stored at −20°C; working concentrations of 5 µM, 10 µM, 50 µM, and 100 µM were prepared. *S. aureus* was stained with various working concentrations of CFDA-SE for 30, 45, and 60 min at 37°C; the optimal concentration and incubation time were standardized and used to assess phagocytosis (37). Purified neutrophils were incubated with stationary phase *L. donovani* promastigotes (virulent AG83 and avirulent DD8) under conditions favoring infection (37°C, 5% CO_2_, 2 h, 5:1 parasite:neutrophil ratio). Following infection and subsequent washing, neutrophils were incubated with CFDA-SE-labeled *S. aureus*, at a 10:1 bacteria:neutrophil ratio for 30 min at 37°C in the dark. Following co-incubation, samples were washed thoroughly to remove non-internalized bacteria, immediately put in ice to stop phagocytosis, and acquired in a flow cytometer. Fluorescence associated with CFDA was quantified as the geomean fluorescence of the neutrophil population, which reflects the relative burden of phagocytosis. Heat-killed *S. aureus* served as a negative control for the assay that demonstrated negligible CFDA geomean fluorescence for the phagocytosis assay.

### Flow cytometry

Neutrophils were gated morphologically based on their characteristic forward and side scatter properties, with fluorescence measured on a bi-exponential scale using BD FACS suite software (BD Biosciences, San Jose, CA, USA). After the initial morphological gating, neutrophils were identified on the basis of CD66b positivity, and the determination of neutrophil dendritic cell hybrids (N-DCs) was done with CD66b^+^/CD83^+^ double positivity (13). The status of co-stimulatory markers CD80 and CD86 was assessed within the N-DC hybrids; 10,000 cells were acquired per tube on a flow cytometer (FACS Verse or FACS Accuri, BD Biosciences, San Jose, CA, USA) and analyzed using BD FACS Suite software (BD Biosciences, San Jose, CA, USA).

A representative figure of the gating strategy was included in Fig. S1A and B. The threshold for all two-dimensional contour/dot plots was established based on quadrant gating of the healthy controls/uninfected neutrophil group, wherein the frequency of CD66b^+^/CD83^+^ populations (upper right quadrant) was negligible. Consequently, for PKDL cases or the infected neutrophil subsets, any observed shift in the upper right quadrant was interpreted as a positive expression, respectively (Fig. S1). As the samples were obtained from field trips, there was a 24-h lag period, and cell morphology was slightly impacted. The lag period between blood collection and processing was maintained at approximately 24 h, wherein blood samples were collected from PKDL patients during active surveillance, primarily between noon to evening. The samples were brought to the lab the following morning and immediately processed; therefore, efforts were made to keep the lag period around 24 h, avoiding a delay of 48 h. Peripheral blood was collected in ethylenediaminetetraacetic acid (EDTA) vials, sealed immediately, with the temperature of collection and transport being 37°C. After sample processing and acquisition, viable neutrophils were observed in the samples, although the amount of cell debris increased, which rose from negligible levels to approximately 5%, when compared with freshly drawn blood. In addition, for assay consistency, 10,000 neutrophils (P1) were gated for each sample analysis. Research supported that no significant differences were observed between neutrophils in stored vs. fresh blood (38). Other studies have indicated that the average lifespan of neutrophils in human circulation was approximately 5.4 days, and not simply ≥24 h, as previously reported (39). Furthermore, neutrophils from both stored (≥24 h) and freshly drawn peripheral blood of healthy volunteers were compared, and no significant differences in viability were observed within the gated P1 neutrophil population (10,000 events). The frequency and expression (GMFC) were evaluated in BD FACS suite (BD Biosciences, San Jose, CA, USA).

### Statistical analysis

The results were expressed as median (interquartile range, IQR) and analyzed using Student’s *t*-test (Unpaired *t*-test for parametric data) and Mann Whitney *t-*test (in case of two groups for non-parametric data) or Kruskal-Wallis test (more than two groups for non-parametric data) using GraphPad Prism software (version 8.4.2, GraphPad Software Inc., La Jolla, CA, USA), with *P* < 0.05 being significant.

## Data Availability

All relevant data are within the article and its supplemental material.

## SUPPLEMENTAL MATERIAL

Fig. S1 iai.00409-24-s0001.tif Gating strategy for identification of CD66b+/CD83+ neutrophils by flow cytometry.

Fig. S2 iai.00409-24-s0002.tif Representative dot plots (A) of Annexin-V positivity and bar graphs (B) indicating the % of apoptosis (early and late apoptosis) in AG83 and DD8 promastigotes (1 x 106/ml) on day 1 and day 9 respectively.

Supplemental material iai.00409-24-s0003.docx Supplemental figure captions.

ASM does not own the copyrights to Supplemental Material that may be linked to, or accessed through, an article. The authors have granted ASM a non-exclusive, world-wide license to publish the Supplemental Material files. Please contact the corresponding author directly for reuse.
