# Supplementary figures and images for: Transdifferentiation of neutrophils facilitates the establishment of infection by Leishmania donovani parasites
Source: Infect Immun. 2025 May 9;93(6):e00409-24. doi: 10.1128/iai.00409-24 (PMC12150689; doi:10.1128/iai.00409-24)

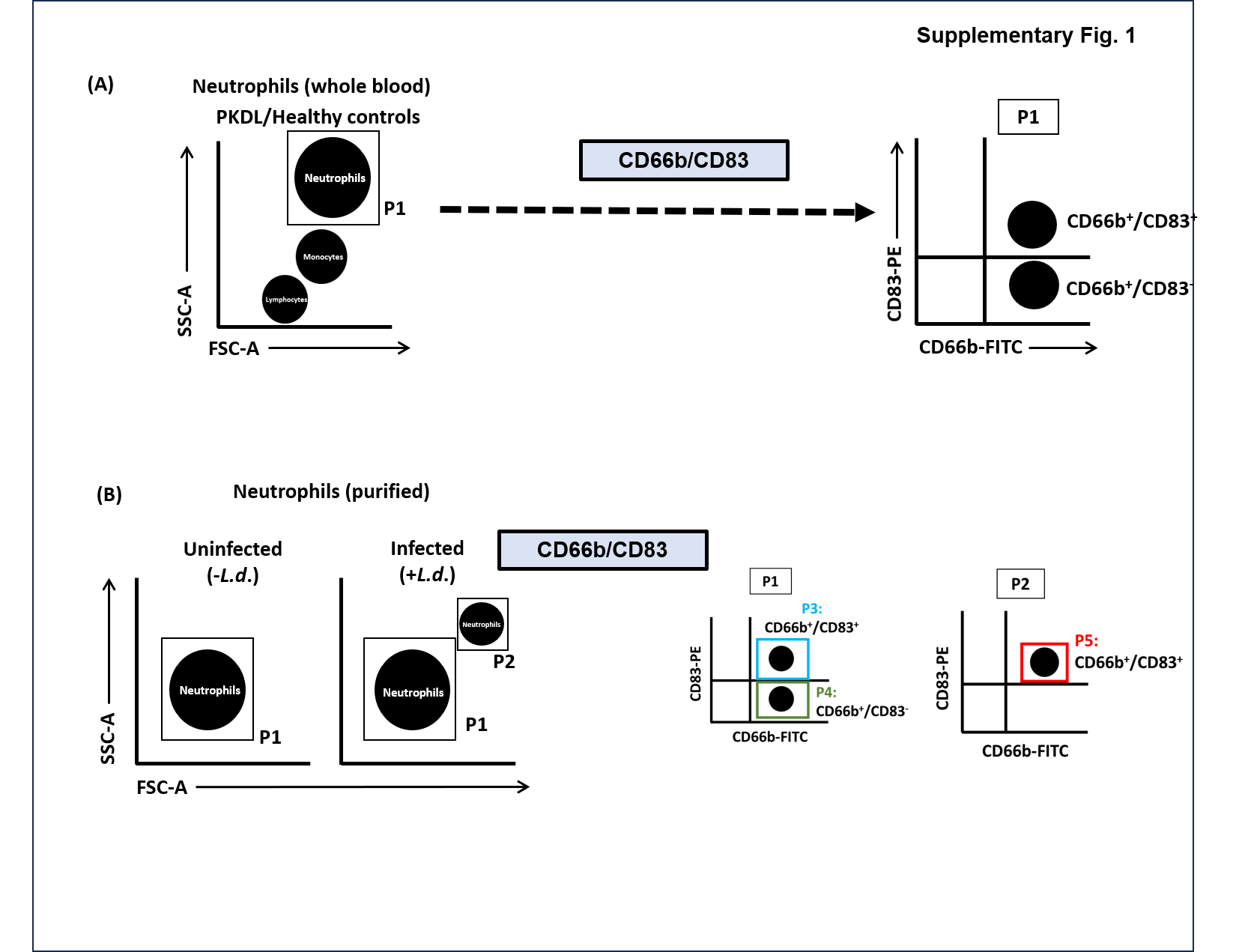

Supplement: Fig. S1 — Gating strategy for identification of CD66b+/CD83+ neutrophils by flow cytometry. [file iai.00409-24-s0001.tif]

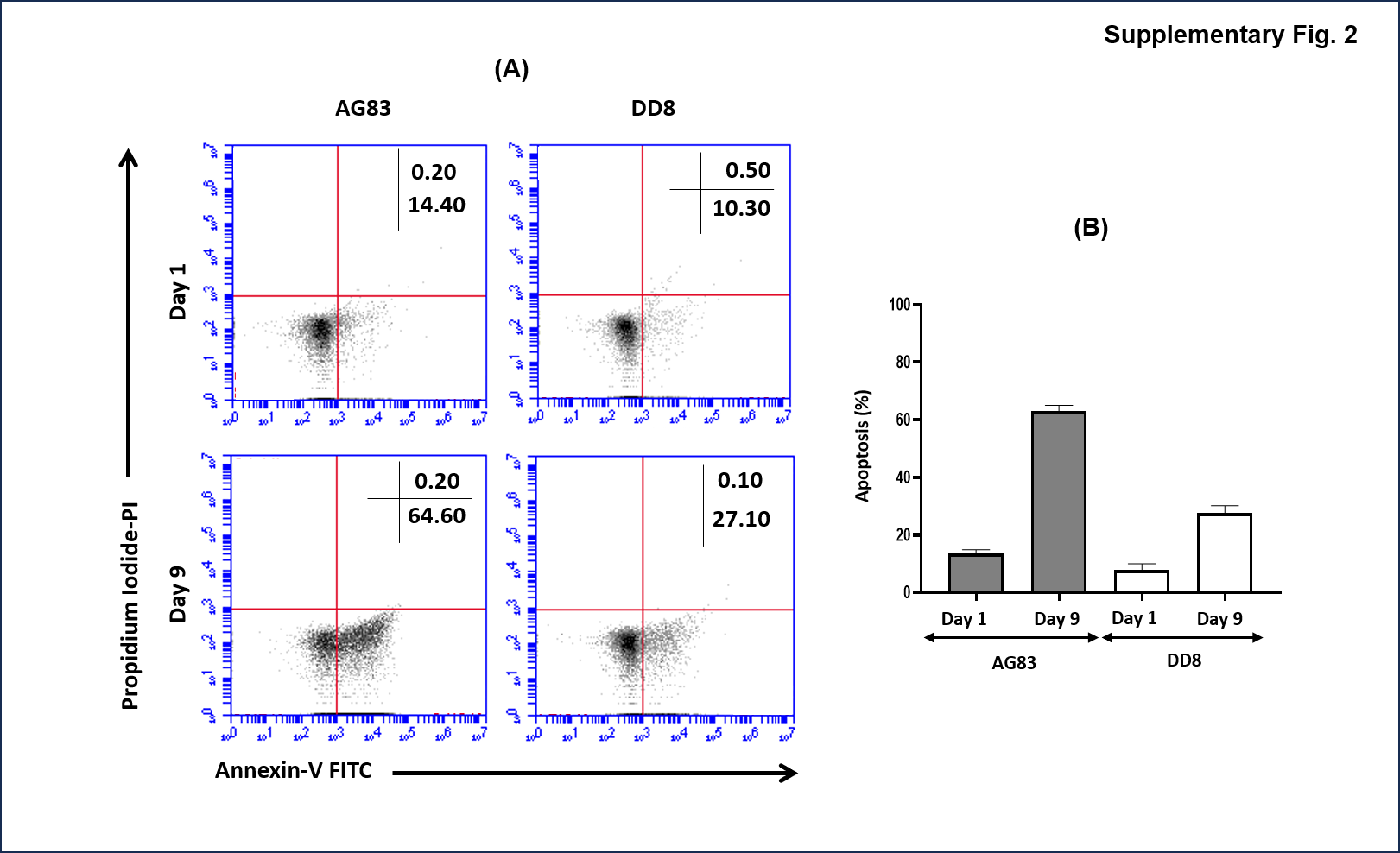

Supplement: Fig. S2 — Representative dot plots (A) of Annexin-V positivity and bar graphs (B) indicating the % of apoptosis (early and late apoptosis) in AG83 and DD8 promastigotes (1 x 10^6^/ml) on day 1 and day 9 respectively. [file iai.00409-24-s0002.tif]
