# Supplementary material for: Transdifferentiation of neutrophils facilitates the establishment of infection by Leishmania donovani parasites
Source: Infect Immun. 2025 May 9;93(6):e00409-24. doi: 10.1128/iai.00409-24 (PMC12150689; doi:10.1128/iai.00409-24)
Supplement: Supplemental material — Supplemental figure captions. [file iai.00409-24-s0003.docx]

Supplementary Fig. 1: Gating strategy for identification of CD66b+/CD83+ 818 neutrophils

by flow cytometry

A: In whole blood, neutrophils were initially identified by morphological gating on forward

scatter area (FSC-A) vs. side scatter area (SSC-A, P1). The neutrophil population P1 was

further confirmed with CD66b positivity, and using two-dimensional contour plots, the

frequency of CD66b+/CD83+ and CD66b+/CD83− 823 subpopulations was obtained.

B: Isolated neutrophils were subjected to ex-vivo infection with L. donovani as described in

materials & methods. Cells were morphologically gated based on FSC-A versus SSC-A and

populations ‘P1’ and ‘P2’ were identified. Subsequently, using CD66b/CD83 positivity, and

two-dimensional dot plots, the P1 population was further sub-grouped into P3

(CD66b+/CD83+)and P4 (CD66b+/CD83−828 ) populations. Additionally, within P2, a P5 gate

(CD66b+/CD83+829 ) was defined.

Supplementary Fig. 2: Status of apoptosis in AG83 and DD8 promastigotes

Representative dot plots (A) of Annexin-V positivity and 832 bar graphs (B) indicating the % of

apoptosis (early and late apoptosis) in AG83 and DD8 promastigotes (1 x 106833 /ml) on day 1

and day 9 respectively.
